# A case of intradiaphragmatic bronchogenic cyst with an abnormally high serum level of CA19‐9

**DOI:** 10.1002/rcr2.838

**Published:** 2021-09-23

**Authors:** Go Kamimura, Kazuhiro Ueda, Soichi Suzuki, Masaya Aoki, Toshiyuki Nagata, Masami Sato

**Affiliations:** ^1^ Department of General Thoracic Surgery Nanphu Hospital Kagoshima Japan; ^2^ Department of General Thoracic Surgery, Graduate School of Medical and Dental Sciences Kagoshima University Kagoshima Japan

**Keywords:** CA19‐9, intradiaphragmatic bronchogenic cyst, intradiaphragmatic tumours

## Abstract

Bronchogenic cysts that occur within the diaphragm are rare and difficult to diagnose preoperatively. We experienced the case of a patient with an abnormally high serum carbohydrate antigen 19‐9 (CA19‐9) level before surgery. The diagnosis of intradiaphragmatic bronchogenic cyst was made at the time of surgery. The patient was a 50‐year‐old woman with upper abdominal pain with an incidentally elevated serum CA19‐9. Although the tumour location could not be established on images, a tumour within the diaphragm was confirmed during the operation. The diaphragm was incised and the tumour was removed together with the capsule. Bronchial cysts were diagnosed histopathologically, and immunohistochemical examination revealed that the bronchial epithelial cells were positive for CA19‐9. When managing patients with bronchogenic cysts in the diaphragm, it is difficult to make a preoperative diagnosis or determine the location of the tumour; thus, careful planning is required before surgery.

## Graphical abstract

Bronchogenic cysts that occur within the diaphragm are rare and difficult to diagnose preoperatively. Here, we report the case of a patient with an abnormally high serum carbohydrate antigen 19‐9 (CA19‐9) level before surgery. The diagnosis of intradiaphragmatic bronchogenic cyst was made during surgery. When managing patients with bronchogenic cysts in the diaphragm, a preoperative diagnosis or location of the tumour can be difficult; thus, careful planning is required before surgery.

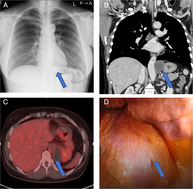

## INTRODUCTION

Bronchogenic cysts that occur within the diaphragm are rare. In such cases, it can be difficult to make a preoperative diagnosis or determine the tumour location.

## CASE REPORT

**FIGURE 1:**
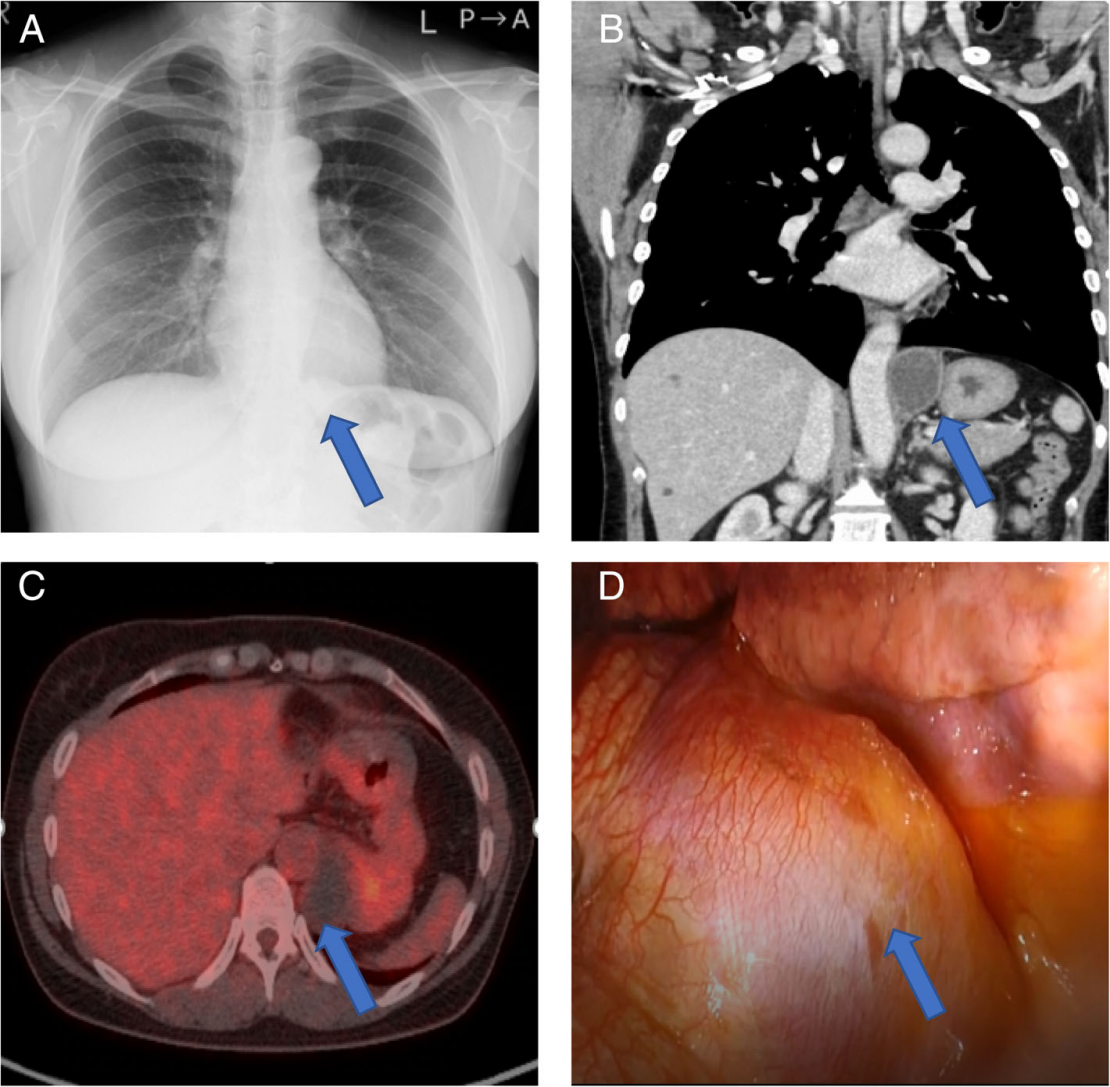
(A) A chest x‐ray showed a nodule near the left diaphragm that overlapped the cardiac shadow. (B) Contrast‐enhanced computed tomography revealed a 5‐cm cystic lesion with a smooth surface and poor contrast effect; however, no clear tumour location could be identified. (C) ^18^F‐fluorodeoxyglucose‐positron emission tomography showed very slight uptake in the tumour. (D) A 5‐cm elevated lesion was found on the dorsal side of the diaphragm

**FIGURE 2:**
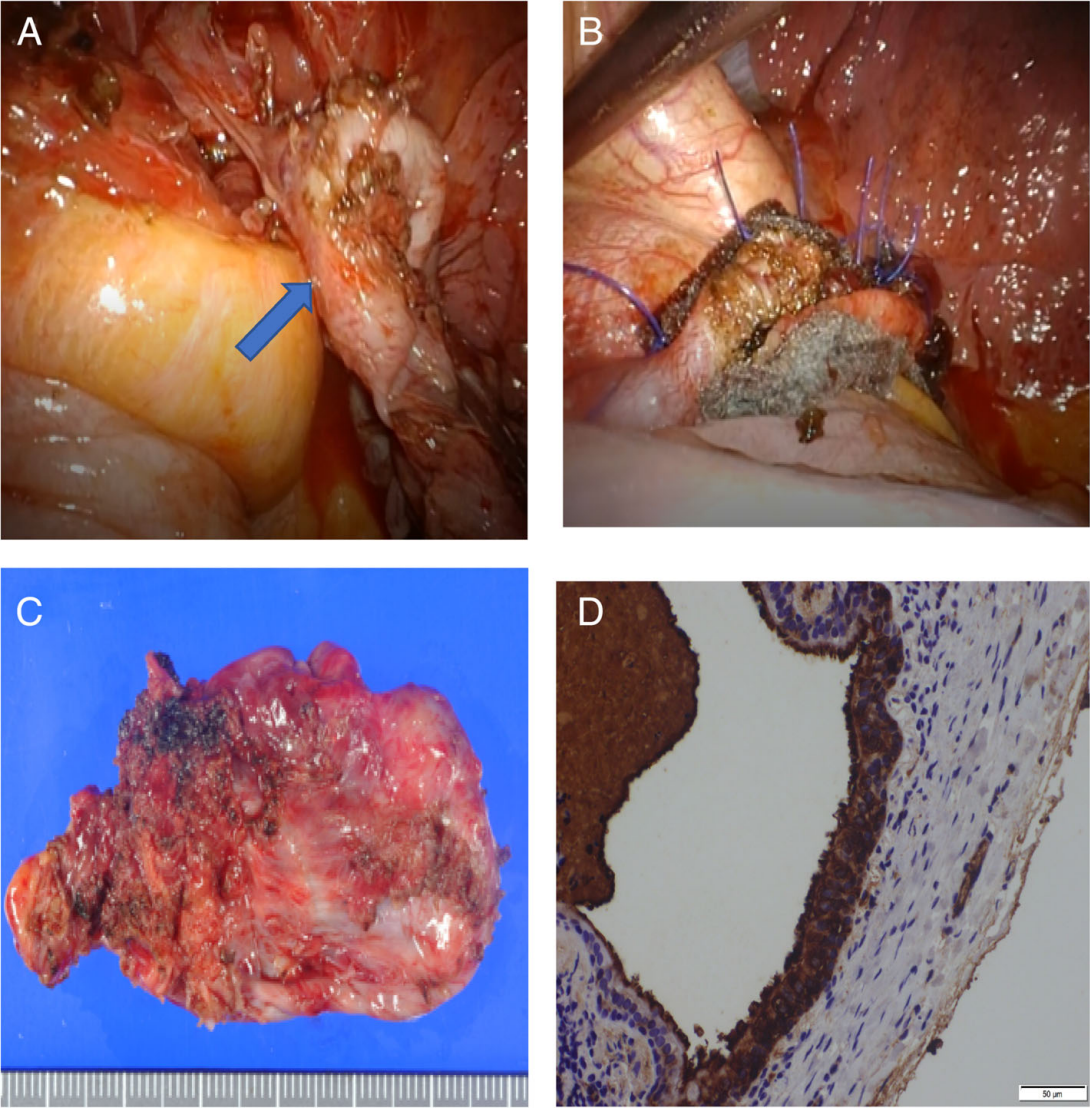
(A) The tumour was removed together with the cyst wall. (B) The diaphragm defect site was repaired by suturing with 2‐0 polypropylene thread using absorbent polyglycolic acid felt (Neoval 03G) strips. (C) Histologically, the cyst wall was covered with bronchial epithelium, and cartilage and bronchial glands were also found, leading to the diagnosis of bronchogenic cyst. (D) Immunostaining demonstrated carbohydrate antigen 19‐9 (CA19‐9) in the bronchial epithelial cells

The patient was a 50‐year‐old woman who had never smoked. She had upper abdominal pain and a high serum carbohydrate antigen 19‐9 (CA19‐9) value (4330.7 U/ml), and a tumour was incidentally detected during further examinations. A chest x‐ray showed a nodule near the left diaphragm that overlapped the cardiac shadow (Figure 1A). Contrast‐enhanced computed tomography (CT) and contrast‐enhanced magnetic resonance imaging (MRI) revealed a 5‐cm cystic lesion with a smooth surface and poor contrast effect; however, accurate tumour location was not identified (Figure 1B). ^18^F‐fluorodeoxyglucose‐positron emission tomography showed only slight uptake in the tumour (Figure 1C). On further close evaluation of the case, bronchogenic cysts, pericardial cysts, oesophageal cysts and similar conditions were proposed as the differential diagnoses. As the tumour size had increased and the tumour marker (CA19‐9) was elevated, we decided to perform surgery. In the left lateral decubitus position, a 2‐ and 3‐cm incisions were made in the eighth intercostal space, and surgery was performed through two ports. A 5‐cm elevated lesion was found on the dorsal side of the diaphragm (Figure 1D). The tumour was located within the diaphragm. When the diaphragm was incised, the cyst wall was partially damaged and white‐like contents were aspirated; however, the tumour was removed together with the cyst wall (Figure 2A). Because the contents had spilled out, the thoracic cavity was irrigated thoroughly after resection of the tumour. Prophylactic antibiotics were given during and after the surgery. The defect of the diaphragm was repaired by suturing with 2‐0 polypropylene thread using strips of absorbent polyglycolic acid felt (Neoveil 03G; Gunze, Osaka, Japan) (Figure 2B). The patient had a good post‐operative course and was discharged on the seventh post‐operative day. The pathological examination revealed a 5‐cm cyst wall (Figure 2C). Histologically, the cyst wall was covered with bronchial epithelium, and cartilage and bronchial glands were also found, leading to the diagnosis of bronchogenic cyst. Immunostaining demonstrated CA19‐9 in bronchial epithelial cells (Figure 2D). Unfortunately, the tumour contents were not submitted for bacterial culture, cytology and chemistry. However, it is probable that the CA19‐9 level in the contents was high. Although the tumour was diagnosed as benign, we planned post‐operative regular check‐ups.

## DISCUSSION

Imaging differentiation between tumours near the diaphragm and within the diaphragm is sometimes difficult. In preoperative examinations, chest CT can only roughly clarify the relationship between a tumour and the surrounding organs, while MRI is often useful if it is used together with CT because the internal intensity values change depending on the properties of the cyst contents.^1^ In many cases, T2‐weighted MRI shows a high signal intensity; however, pericardial cysts, oesophageal cysts, neurogenic tumours and lymphomas are differentiated based on the tumour location, and the diagnosis is rarely confirmed by imaging alone. As the diaphragm is only several millimetres thick, it is often difficult to distinguish whether the tumour is located on the diaphragm, within the diaphragm or under the diaphragm. It is therefore necessary to prepare an approach that can be applied to any part of the diaphragm before surgery. According to a previous review by Mubang et al., 21 cases of intradiaphragmatic bronchogenic cyst were reported between 1955 and 2015.^2^ These cases included nine males and 12 females. The average age was 42.7 years, 71.4% had left‐sided occurrence and 14% had no symptoms. Intradiaphragmatic bronchogenic cysts were resected via open thoracotomy in 47.4% of cases, followed by open laparotomy in 15.8%, both thoracotomy and laparotomy in 15.8%, thoracoscopy in 15.8% and laparoscopy in 5.2%. The diaphragm was repaired by direct suture in 69.2% of cases and by patch plasty in 23.1%. The tumour location was misdiagnosed in 61.9% of patients; the majority were misdiagnosed as either posterior mediastinal tumours or adrenal tumours. Although percutaneous or transbronchial aspiration cytology has been reported to be useful,^3^ the risk of infection by puncture, especially in areas that are difficult to puncture due to respiratory movement, must be considered. In rare cases, bronchogenic cysts have been reported to become malignant,^4^ and as with mediastinal tumours (e.g., thymic epithelial tumours), biopsy should not be performed if it is judged to be resectable. Bronchogenic cysts are classified into intrapulmonary and mediastinal types, most of which are asymptomatic, but intrapulmonary types sometimes have communication with the bronchi and may be symptomatic. It is transmitted through the respiratory tract, and when drainage becomes difficult, it becomes difficult to treat. In the case of the mediastinal type, the risk of infection is not as high as in the intrapulmonary type; however, if infected, it causes mediastinitis, which is a serious complication.^1^ With infection, inflammatory adhesion to the surroundings makes complete resection of the cyst wall difficult. It tends to increase in the long term, so it is desirable to remove before it becomes complicated by infection.

CA19‐9 is mainly elevated in gastrointestinal cancers (e.g., pancreatic cancer and cholangiocarcinoma) and inflammatory diseases (e.g., cholangitis). It is also present in normal cells, bronchial glands and bile ducts, and CA19‐9 levels in intrapulmonary bronchogenic cysts have been reported to be high.^5^ In this case, the cyst occurred in the diaphragm, but the CA19‐9 level was high. Because intradiaphragmatic bronchogenic cysts are difficult to diagnose preoperatively, the measurement of CA19‐9 may be useful to confirm the diagnosis.

## CONFLICT OF INTEREST

None declared.

## AUTHOR CONTRIBUTION

Soichi Suzuki, Masaya Aoki and Toshiyuki Nagata participated in the operation of this case. Masami Sato supervised the operation and the editing of the manuscript. Kazuhiro Ueda drafted the manuscript, and all authors read and approved the final manuscript.

## ETHICS STATEMENT

Appropriate written informed consent was obtained for publication of this case report and accompanying images.
